# Ergogenic effects of spinal cord stimulation on exercise performance following spinal cord injury

**DOI:** 10.3389/fnins.2024.1435716

**Published:** 2024-08-29

**Authors:** Daniel D. Hodgkiss, Alison M. M. Williams, Claire S. Shackleton, Soshi Samejima, Shane J. T. Balthazaar, Tania Lam, Andrei V. Krassioukov, Tom E. Nightingale

**Affiliations:** ^1^School of Sport, Exercise and Rehabilitation Sciences, University of Birmingham, Birmingham, United Kingdom; ^2^International Collaboration on Repair Discoveries, University of British Columbia, Vancouver, BC, Canada; ^3^School of Kinesiology, University of British Columbia, Vancouver, BC, Canada; ^4^Division of Physical Medicine and Rehabilitation, Department of Medicine, University of British Columbia, Vancouver, BC, Canada; ^5^Department of Rehabilitation Medicine, University of Washington, Seattle, WA, United States; ^6^Division of Cardiology, Department of Echocardiography, Vancouver General and St. Paul’s Hospital, University of British Columbia, Vancouver, BC, Canada; ^7^GF Strong Rehabilitation Centre, Vancouver Coastal Health, Vancouver, BC, Canada

**Keywords:** spinal cord injuries, spinal cord stimulation, autonomic nervous system, exercise performance, cardiovascular control

## Abstract

Cervical or upper-thoracic spinal cord injury (SCI, ≥T6) often leads to low resting blood pressure (BP) and impaired cardiovascular responses to acute exercise due to disrupted supraspinal sympathetic drive. Epidural spinal cord stimulation (invasive, ESCS) and transcutaneous spinal cord stimulation (non-invasive, TSCS) have previously been used to target dormant sympathetic circuits and modulate cardiovascular responses. This case series compared the effects of cardiovascular-optimised ESCS and TSCS versus sham ESCS and TSCS on modulating cardiovascular responses and improving submaximal upper-body exercise performance in individuals with SCI. Seven males with a chronic, motor-complete SCI between C6 and T4 underwent a mapping session to identify cardiovascular responses to spinal cord stimulation. Subsequently, four participants (two ESCS and two TSCS) completed submaximal exercise testing. Stimulation parameters (waveform, frequency, intensity, epidural electrode array configuration, and transcutaneous electrode locations in the lumbosacral region) were optimised to elevate cardiovascular responses (CV-SCS). A sham condition (SHAM-SCS) served as a comparison. Participants performed arm-crank exercise to exhaustion at a fixed workload corresponding to above ventilatory threshold, on separate days, with CV-SCS or SHAM-SCS. At rest, CV-SCS increased BP and predicted left ventricular cardiac contractility and total peripheral resistance. During exercise, CV-SCS increased time to exhaustion and peak oxygen pulse (a surrogate for stroke volume), relative to SHAM-SCS. Ratings of perceived exertion also tended to be lower with CV-SCS than SHAM-SCS. Comparable improvements in time to exhaustion with ESCS and TSCS suggest that both approaches could be promising ergogenic aids to support exercise performance or rehabilitation, along with reducing fatigue during activities of daily living in individuals with SCI.

## Introduction

Spinal cord injury (SCI) is a multifaceted neurological condition affecting over 20 million people worldwide (GBD Spinal Cord Injuries Collaborators, 2023), resulting in a loss of sensory, motor, and autonomic functions below the level of the lesion. The majority of individuals with a motor-complete cervical or upper-thoracic (i.e., above the sixth thoracic segment; >T6) SCI typically exhibits cardiovascular dysfunction resulting from disrupted pathways between the medulla oblongata and the sympathetic pre-ganglionic neurons at and below the level of injury (West et al., 2015; Partida et al., 2016; Cruz and Blauwet, 2018). During exercise, diminished supraspinal sympathetic output to the heart and blood vessels is thought to reduce peak heart rate (HR), lower brachial blood pressure (BP), venous return and thus stroke volume (SV), and ultimately limit exercise performance (Claydon et al., 2006; West et al., 2012; Schmid et al., 1998; Bhambhani, 2002). Overtime, a reduction in habitual physical activity (Nightingale et al., 2017; van den Berg-Emons et al., 2010), particularly of a sufficient intensity (Bhambhani, 2002; Cowley, 2018), results in accelerated physical deconditioning that is associated with low levels of cardiorespiratory fitness (CRF) (Haisma et al., 2006; Simmons et al., 2014). This physical deconditioning represents a model of advanced ageing whereby the risk of developing chronic conditions such as cardiovascular disease, stroke, cognitive impairment, and type-2 diabetes mellitus is likely to occur at an earlier age and also at a heightened frequency (Bauman et al., 1999; Groah et al., 2012). This risk is also significantly elevated with a low CRF (Wu et al., 2012; Cragg et al., 2013), thereby increasing the risk of mortality (Cragg et al., 2013). Moreover, individuals with a higher neurological level of injury are at a greater risk for developing such health complications (Groah et al., 2001).

To overcome the impaired cardiovascular responses during exercise, various strategies to augment the cardiovascular system in individuals with SCI have been explored [e.g., abdominal binding (Kerk et al., 1995; West et al., 2014), lower-body positive pressure (Pitetti et al., 1994), compression garments (Rimaud et al., 2007), and α − 1 agonist midodrine (Nieshoff et al., 2004)], yet the evidence on their effectiveness to enhance acute upper-body exercise performance is inconclusive. Therefore, individuals, particularly athletes, are known to intentionally induce autonomic dysreflexia (AD) to elicit a sympathetic response and increase BP during exercise. This practice is called “boosting” (Gee et al., 2015, 2021) and has been shown to increase peak oxygen uptake (V̇O_2peak_) by 6–24% (Burnham et al., 1994; Schmid et al., 2001; Nightingale et al., 2022) and therefore provides individuals with SCI with an ergogenic advantage. Despite the potential benefits, there are life-threatening consequences associated with such large and uncontrolled elevations in BP (Wan and Krassioukov, 2014).

Spinal cord stimulation (SCS) has received overwhelming attention in recent years for targeting the recovery of various neurological dysfunctions. Epidural SCS (ESCS) involves the activation of dorsal root afferents using a surgically implanted electrode array on top of the dura that is connected to a pulse generator. Early research demonstrated that ESCS paired with activity-based therapy could improve walking speed and lower perception of effort in individuals with motor-incomplete SCI (Herman et al., 2002; Carhart et al., 2004; Ganley et al., 2005). Recently, studies have shown that ESCS can effectively modulate cardiovascular output in individuals with motor-complete SCI (Harkema et al., 2018; Aslan et al., 2018; West et al., 2018; Samejima et al., 2023) and can increase V̇O_2peak_ by up to 26% during an upper-body exercise test to exhaustion in an individual with motor-complete tetraplegia (Nightingale et al., 2019). This acute improvement drastically outweighs the average 9% increase in V̇O_2peak_ achieved following 10–37 weeks of upper-body aerobic exercise training in individuals with tetraplegia (Figoni et al., 2021). The use of ESCS may also obviate the need to use a practice such as boosting to enhance exercise performance. Despite its promising potential, the accessibility of ESCS within the SCI community is limited by its invasive surgical procedure (Taccola et al., 2020) and expensive cost (Shipley and North, 2018). Alternatively, non-invasive transcutaneous SCS (TSCS) can be used to target the same dorsal afferents as ESCS without surgical intervention (Hofstoetter et al., 2018; Ladenbauer et al., 2010; Danner et al., 2011). While it has been demonstrated that TSCS can target cardio-autonomic dysfunction, such as the mitigation of orthostatic hypotension (Phillips et al., 2018), its effectiveness for improving cardiovascular responses during acute, submaximal exercise is currently unknown. In comparison with ESCS, this approach may be safer and could offer greater flexibility in positioning electrodes to target cardiovascular function, and thus be used as a combined exercise strategy in the future.

This study aimed to compare the acute effects of ESCS and TSCS (relative to sham conditions) on modulating resting cardiovascular responses (e.g., BP, SV, and cardiac contractility) and exercise performance, assessed as time to exhaustion during submaximal upper-body exercise, in individuals with SCI. We hypothesised that there would be comparable improvements in time to exhaustion with both ESCS and TSCS when optimised to improve cardiovascular responses, relative to sham conditions.

## Materials and methods

This study received ethical approval from the University of British Columbia Clinical Research Ethics Board (H19-00932) and was conducted in accordance with the Declaration of Helsinki. Testing was performed at the International Collaboration on Repair Discoveries in the Blusson Spinal Cord Centre, University of British Columbia, Vancouver, Canada.

**Figure 1:**
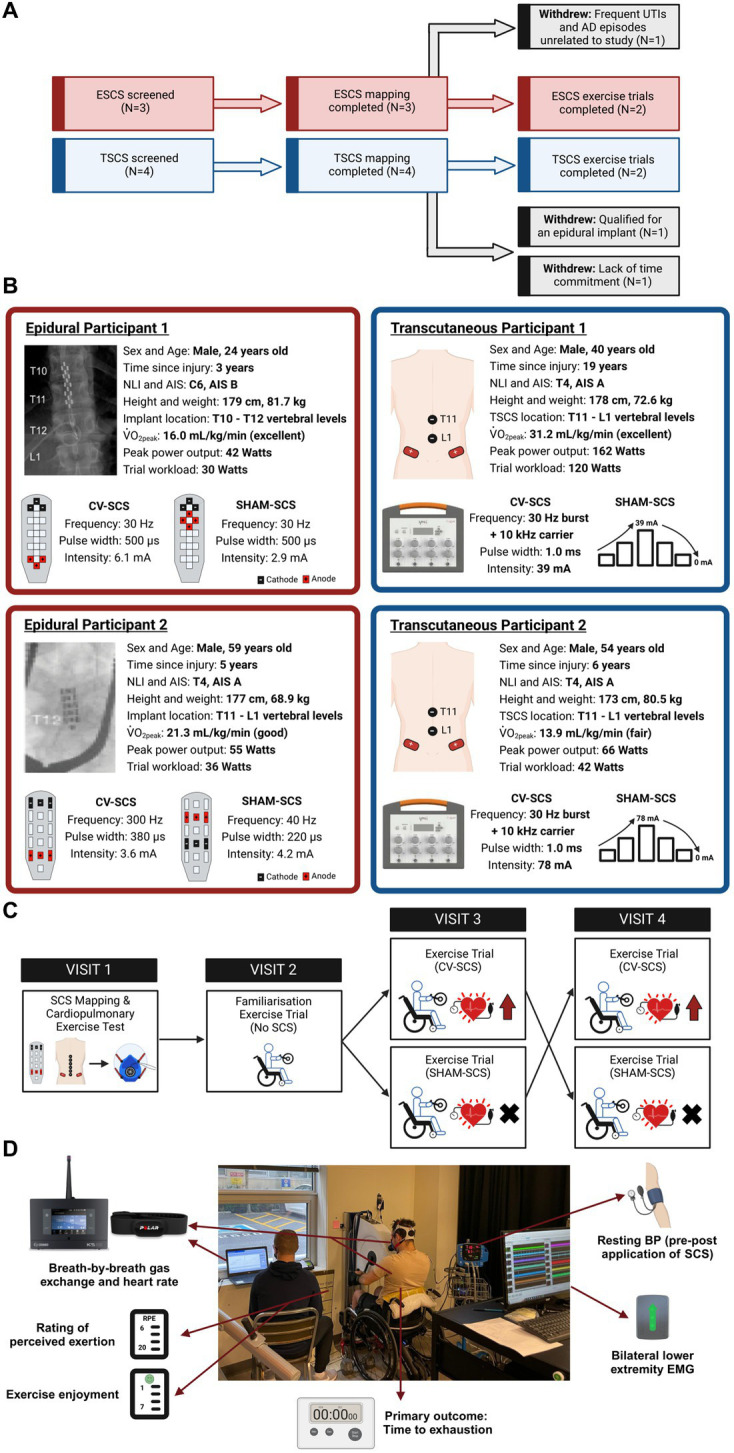
**(A)** Flow diagram of participants throughout the study. Three participants completed the mapping session but withdrew due to reasons unrelated to the study (grey-shaded boxes). **(B)** Participant demographics, injury characteristics and trial workloads for the two epidural (ESCS P1 and P2) and two transcutaneous participants (TSCS P1 and P2). Conventional X-rays highlight the positions of the 16-electrode arrays in the two epidural participants. Parameters for cardiovascular-optimised spinal cord stimulation (CV-SCS) and sham spinal cord stimulation (SHAM-SCS) are presented for all participants, along with specific epidural electrode configurations and transcutaneous electrode locations (black, cathodes; red, anodes). The ESCS frequencies used for the participants herein align with the broad range of frequencies used by participants elsewhere for volitional and autonomic functions (i.e., 18–700 Hz) (Pino et al., 2022). Classifications of peak oxygen uptake (V̇̇O_2peak_) are based on reference fitness values for the SCI population, as described by Simmons et al. (2014). Participants demonstrated a range of fitness levels (i.e., fair to excellent). Demographics of the three participants who completed the mapping session but withdrew prior to the exercise trials are presented in Supplementary material S1. **(C)** Schematic of the study design. Participants underwent a spinal cord stimulation (SCS) mapping session followed by a cardiopulmonary exercise test on an arm-crank ergometer to volitional exhaustion to identify V̇̇O_2peak_ and ventilatory threshold (V_T_). Following a familiarisation trial, participants performed two exercise trials in a randomised order, with CV-SCS or SHAM-SCS. Trials were performed to exhaustion at a fixed workload corresponding to the above V_T_. All visits were separated by at least 3 days. **(D)** Overview of outcome measures during the time to exhaustion exercise trials. AIS, American Spinal Injury Association Impairment Scale; Hz, hertz; kHz, kilohertz; mA, milliamp; NLI, neurological level of injury; TSCS, transcutaneous spinal cord stimulation; μs, microseconds.

Participants were required to meet the following inclusion criteria: (1) aged 18–65 years; (2) non-progressive SCI ≥ T6; (3) ≥1-year post-injury; (4) motor-complete SCI [American Spinal Injury Association Impairment Scale (AIS) A-B, determined via the 2019 version of International Standards for Neurological Classification of Spinal Cord Injury examination (Rupp et al., 2021)]; (5) no musculoskeletal dysfunction, unhealed fractures, pressure sores, or active infections; (6) no cardiovascular, respiratory, bladder, or renal disease unrelated to the SCI; and (7) no additional implanted medical devices (e.g., baclofen pumps and pacemakers). Seven males provided written informed consent to participate in this study. Three participants were recruited who were already implanted with a 16-electrode array (Restore-ADVANCED neurostimulator, Specify 5-6-5, Medtronic, Minneapolis, MN, United States) between the T10 and T12 spinal segments at least 7 months prior to the study. Four participants were not implanted with an epidural stimulator and therefore received TSCS. Three participants completed the mapping session but withdrew from the study prior to the exercise trials for reasons unrelated to the study protocol (demographics are provided in Supplementary material S1). Therefore, two ESCS and two TSCS participants completed the study (Figure 1).

### Study design and assessments

**Figure 2:**
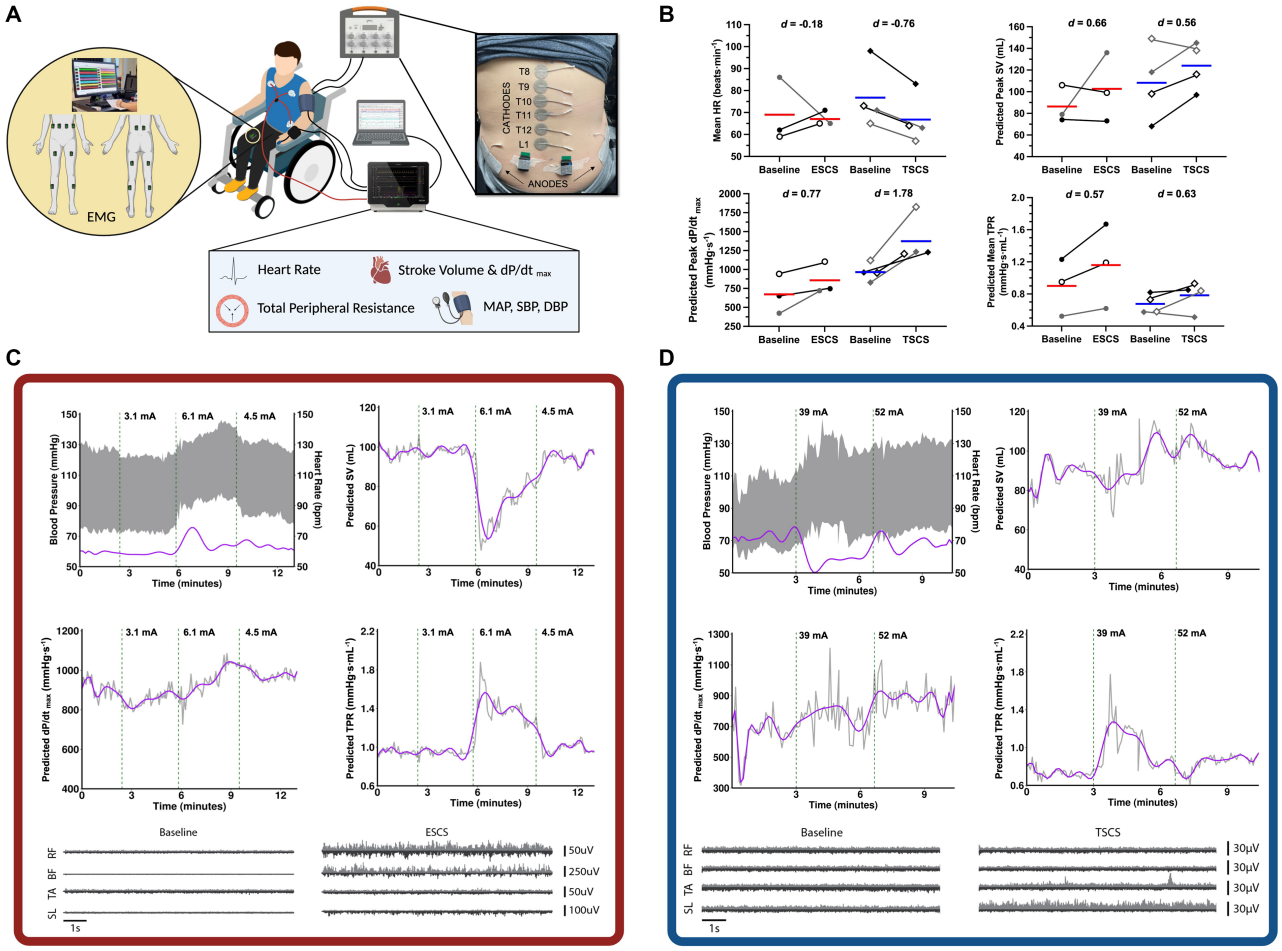
Mapping session for the selection of cardiovascular-optimised (CV-SCS) and sham spinal cord stimulation (SHAM-SCS) parameters. **(A)** An example mapping session setup with beat-by-beat blood pressure (BP) and heart rate (HR) recorded via finger plethysmography and electrocardiogram. Surface electromyography (EMG) recordings from trunk (RA, EO, and ES) and lower limb musculature (BF, RF, MG, and TA). **(B)** Pooled cardiovascular responses at baseline and with CV-SCS, including mean HR, predicted stroke volume (SV), left ventricular contractility (dP/dt_max_), and total peripheral resistance (TPR). Red and blue bars represent the mean for ESCS and TSCS participants, respectively. Individual data are presented in black for two ESCS participants (ESCS P1, open circles; ESCS P2, closed circles) and two TSCS participants (TSCS P1, open diamonds; TSCS P2, closed diamonds). Individual data are also presented in grey for the one ESCS participant (ESCS P3, closed circles) and the two TSCS participants (TSCS P3, open diamonds; TSCS P4, closed diamonds) who withdrew from the study prior to performing the exercise trials. Data for all participants are provided as Supplementary material S1. Effect sizes are reported as Cohen’s *d*, with >0.2, >0.5 and > 0.8 representing small, moderate and large effects, respectively. Representative cardiovascular responses with and without CV-SCS are demonstrated for an ESCS participant **(C)** and a TSCS participant **(D)**. Representative lower extremity (RF, BF, TA, and SL) EMG recordings are also shown while the participants were at rest with and without CV-SCS. The top trace (light grey) of each plot represents the right muscle, while the bottom trace (dark grey) represents the left muscle. BF, biceps femoris; EO, external obliques; ES, erector spinae; ESCS, epidural spinal cord stimulation; RA, rectus abdominis; RF, rectus femoris; MG, medial gastrocnemius; SL, soleus; TA, tibialis anterior; TSCS, transcutaneous spinal cord stimulation.

During the first visit, participants underwent an ESCS or TSCS mapping session to identify the specific stimulation parameters (i.e., waveform, frequency, intensity, specific configurations, and electrode locations over specific spinal processes) to be used during the exercise trials (Figure 2). Beat-by-beat BP and HR were recorded via finger plethysmography and electrocardiogram (Finapres NOVA, Finapres Medical Systems, Amsterdam, Netherlands) and sampled at 1000 Hz through an analog-to-digital converter (Powerlab 16/35 System, AD Instruments, Colorado Springs, United States). Beat-by-beat BP was corrected using episodic brachial BP measurements every minute (Dinamap Pro, GE Healthcare, Chicago, United States) via LabChart 8 (AD Instruments, Colorado Springs, United States). SV, cardiac contractility, and total peripheral resistance (TPR) were predicted via the integrated Modelflow^®^ algorithm. Throughout the mapping session, motor activity was recorded via surface electromyography (EMG) sensors (Trigno, Delsys Inc., Boston, United States). Sensors were affixed bilaterally to the rectus abdominis, external obliques, erector spinae at the L4 level, biceps femoris, rectus femoris, tibialis anterior, and medial head of the gastrocnemius. Lower extremity muscle activity was recorded to assess whether any elevations in BP could be due to the reactivation of the skeletal muscle pump. All EMG data were collected at 2000 Hz and stored for offline analysis. Further information on the processing and analysis of the EMG data is provided as Supplementary material S2. ESCS was manipulated by altering the frequency, intensity, and pulse widths of several pre-specified electrode configurations, as programmed by the manufacturer [Verita Neuro (ESCS P1); Tripole™ and Proclaim elite™, Abbott (ESCS P2)]. TSCS was delivered using an isolated constant current stimulator (TESCoN, SpineX Inc., California, United States). Stimulation was applied via 2.5 cm self-adhesive round cathode electrodes placed on the skin at the midline over the vertebral column, and two 5.0 × 10.2 cm^2^ rectangular anode electrodes placed bilaterally on the skin over the iliac crests. The stimulation consisted of a high carrier frequency pulse (10 kHz) overlapping charge-balanced monophasic rectangular waveforms at 30 Hz burst frequency, each with a 1.0 ms pulse width.

Stimulation parameters were chosen for two conditions: (1) those that achieved a gradual, optimal, and controlled elevation in BP without discomfort, symptoms, or exceeding the 150 mmHg cutoff for pharmacological-mediated AD intervention (Paralyzed Veterans of America Consortium for Spinal Cord Medicine, 1997) [i.e., cardiovascular-optimised SCS (CV-SCS)] and (2) those that did not alter cardiovascular responses (SHAM-SCS). For the ESCS sham condition, specific electrode configurations were chosen that did not modulate BP. For the TSCS sham condition, the stimulation intensity was increased to sensory threshold and slowly decreased to 0 mA prior to the start of the exercise trial (Estes et al., 2021). Representative traces for an ESCS and a TSCS participant, along with pooled data across all seven participants, are presented in Figure 2.

Following the mapping session, participants performed a graded cardiopulmonary exercise test (CPET) to volitional exhaustion on an electronically braked arm-crank ergometer (Angio CPET, Lode B.V., Groningen, Netherlands). Following a 3 min warm-up, workload was increased by 4–10 watts (W) per minute. These increments were chosen by a trained clinical exercise physiologist and were based on differing neurological levels of injury to facilitate volitional exhaustion within 8–12 min. Participants were advised to cycle at approximately 70 revolutions per minute (rpm) and were given consistent encouragement throughout. The test was terminated upon volitional exhaustion, defined as a drop in cadence below 30 rpm for a total of three times, or for more than 10 s on one occasion. Breath-by-breath gas exchange variables (K5, COSMED, Rome, Italy) and HR (Garmin Dual, Garmin, Olathe, KS, United States) were recorded continuously. Ratings of perceived exertion (RPE) were recorded at the end of each minute using the Borg 6–20 scale (Borg, 1982). V̇O_2peak_ was determined as the highest 15-breath rolling average attained during the test. Attainment of V̇O_2peak_ required meeting two of the following three criteria: (1) plateau in V̇O_2_, (2) respiratory exchange ratio (RER) ≥1.15, and (3) RPE ≥19 (Alrashidi et al., 2022). Ventilatory thresholds (V_T_) were identified using a variety of strategies and via an online application tool (Gaskill et al., 2001; Keir et al., 2022). All thresholds were agreed upon by two independent raters and enabled the identification of a workload (calculated using the V̇O_2peak_ data at the time upon which each V_T_ occurred) corresponding to either V_T2_ (prescribed for ESCS P1 and TSCS P1) or the midpoint between V_T1_ and V_T2_ (prescribed for ESCS P2 and TSCS P2).

### Exercise trials

A familiarisation trial was conducted without SCS to cross-check the prescribed exercise intensity. Participants were then randomised to perform two exercise trials to exhaustion, separated by at least 3 days of recovery, with CV-SCS and SHAM-SCS. Participants arrived at the laboratory following a 4 h fast, having also refrained from caffeine consumption for >12 h, and avoided alcohol consumption and vigorous-intensity exercise for >24 h. Participants performed a thorough bowel routine in the preceding 24 h. The ESCS participants turned off their devices at least 3 h prior to arrival to prevent any residual effects of the stimulation during the trials. Upon arrival, participants emptied their bladder, had their weight recorded, and answered pre-exercise readiness questions to assess their health status (i.e., absence of flu-like symptoms, urinary tract infection, or pressure sores). All trials were performed at a similar time of day (± 2 h) to avoid circadian rhythm effects. Three resting brachial BP measurements were taken (Dinamap Pro, GE Healthcare, Chicago, IL, United States) before and after an unblinded clinician adjusted the stimulation parameters (via the TESCoN device or the participant’s epidural neurostimulator device) as per the trial allocation (CV-SCS or SHAM-SCS). The participants and the remaining researchers were blinded to the trial randomisation. Following a 5 min warm-up at 5 W, participants exercised at a constant workload corresponding to their V_T_ (see Figure 1 for each participant’s workloads). On occasions where participants demonstrated no signs of fatiguing by the 40 min timepoint, the intensity was increased to a work rate corresponding to the next physiological threshold or beyond (i.e., V_T2_, +10% V_T2_, +20% V_T2_) (Nelson et al., 2014). The primary outcome was time to exhaustion (mm:ss). Breath-by-breath gas exchange variables were recorded via a facemask attached to a portable metabolic cart (K5, COSMED, Rome, Italy), and HR was recorded continuously via the Polar Beat application (Polar H10, Polar Electro, Kempele, Finland). Oxygen uptake and HR were used to calculate oxygen pulse (a reasonable surrogate for SV). The participant’s RPE (Borg 6–20 scale) and exercise enjoyment (1–7 scale, whereby 1 = *not at all* and 7 = *extremely*) (Stanley et al., 2009) were recorded in the final 15 s of each 5 min interval. Trunk and lower extremity muscle activity was recorded via surface electromyography (EMG) sensors (Trigno, Delsys Inc., Boston, United States). However, we were unable to effectively filter TSCS artefacts from the EMG signals recorded at the trunk for subsequent analysis, so only EMG results from the legs are reported herein. Further information is provided as Supplementary material S2. Superficial femoral artery blood flow and vessel diameter (5.2–13 MHz, Vivid 7, GE Healthcare, Mississauga, ON) were assessed in one matched SCS pair (ESCS P2 and TSCS P2) (Supplementary Figure S1). Brachial BP was recorded in triplicate immediately post-exercise.

### Data analysis

Data are presented as means with individual data points. Effect sizes are reported as Cohen’s *d*, with *d* > 0.2, *d* > 0.5, and *d* > 0.8 representing small, moderate, and large effects, respectively (Cohen, 1988).

## Results

**Figure 3:**
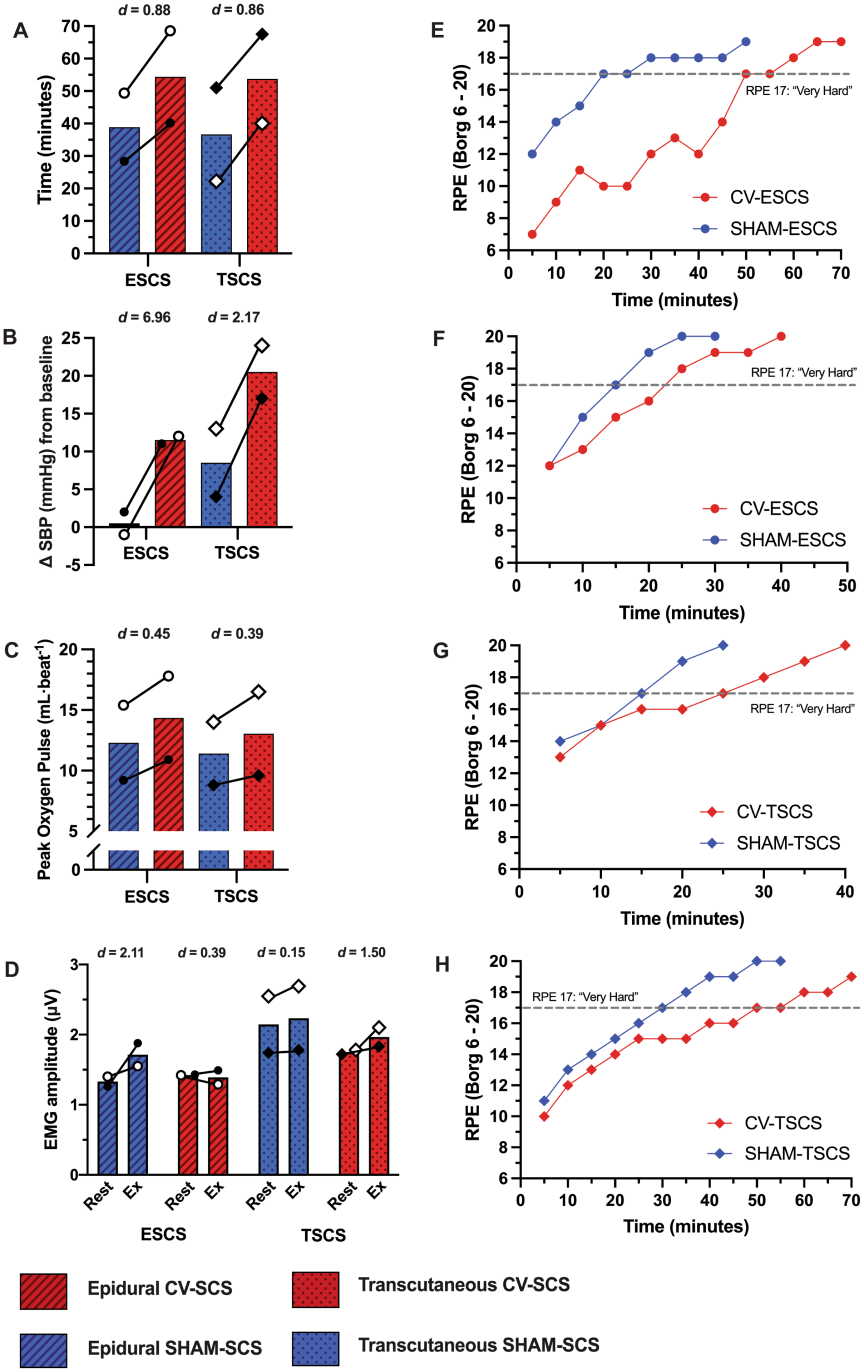
Cardiorespiratory data during the time to exhaustion exercise trials with cardiovascular-optimised (CV-SCS) and sham spinal cord stimulation (SHAM-SCS). Individual data points are presented for each epidural (ESCS P1, open circles; ESCS P2, closed circles) and transcutaneous participants (TSCS P1, open diamonds; TSCS P2, closed diamonds). Time to exhaustion **(A)**, change in systolic blood pressure (SBP) prior to exercise **(B)**, and peak oxygen pulse **(C)** were all greater with CV-SCS, relative to SHAM-SCS, in both ESCS and TSCS participants. **(D)** Despite a range of small to large effect sizes, electromyography (EMG) data demonstrate negligible changes in leg muscle activity from rest to during exercise with CV-SCS and SHAM-SCS. Ratings of perceived exertion (RPE) are presented for each ESCS participant trial [ESCS P1 **(E)**, ESCS P2 **(F)**, TSCS P1 **(G)**, and TSCS P2 **(H)**]. RPE was lower throughout the exercise trials with CV-SCS, and it took longer for participants to perceive the exercise to be “very hard” (RPE of 17, grey dashed line) relative to SHAM-SCS.

Data from the mapping sessions indicate that with optimised stimulation parameters, ESCS and TSCS increased resting BP and predicted left ventricular cardiac contractility and TPR (Figure 2). During the exercise trials, time to exhaustion was greater with CV-SCS in all participants, relative to SHAM-SCS (Figure 3A), and was similar between ESCS and TSCS. The change in systolic BP at rest was larger with CV-SCS relative to SHAM-SCS (Figure 3B). Peak oxygen pulse was greater with CV-SCS in comparison with SHAM-SCS (Figure 3C). Overall, leg muscle activity was similar between rest and exercise in both SHAM-SCS and CV-SCS (Figure 3D). Average leg muscle activity increased no more than 1 μV between conditions for any participant, and the average increase in activity was only 0.16 μV. Relative to SHAM-SCS, RPE tended to be lower with CV-SCS at each time point despite a matched workload prescribed across trials (Figures 3E–H). Peak systolic velocity and shear rate were greater with CV-SCS, relative to SHAM-SCS (Supplementary Figure S1). Values for these comparisons at rest and during the exercise trials are reported in Table 1. There were no adverse events related to the exercise protocol, ESCS or TSCS, but one ESCS participant developed a skin lesion on their back due to friction with their chair unrelated to the study.

**Table 1:** Individual time to exhaustion, cardiorespiratory, and psychophysiological data for each epidural and transcutaneous participant at rest and during the exercise trials performed with cardiovascular-optimised (CV-SCS) and sham spinal cord stimulation (SHAM-SCS).

	ESCS P1		ESCS P2		ESCS *d*	TSCS P1		TSCS P2		TSCS *d*
	CV-SCS	SHAM-SCS	CV-SCS	SHAM-SCS		CV-SCS	SHAM-SCS	CV-SCS	SHAM-SCS	
***Rest***										
SBP without SCS (mmHg)	104	100	134	129	0.22	118	130	107	105	0.37
DBP without SCS (mmHg)	53	55	68	62	0.24	70	72	65	57	0.38
ΔSBP with SCS (mmHg)	12	-1	11	2	6.96	24	13	17	4	2.17
***Exercise***										
Time to exhaustion (mm:ss)	68:35	49:24	40:11	28:24	0.88	40:04	22:15	67:30	51:02	0.86
SBP post-exercise (mmHg)	124	101	132	107	4.71	125	136	140	125	0.21
O_2_ pulse (mL/beat)	13.7	12.1	7.7	6.7	0.32	13.0	12.0	8.5	7.9	0.27
Peak O_2_ pulse (mL/beat)	17.8	15.4	10.9	9.2	0.45	16.5	14.0	9.6	8.8	0.39
HR (bpm)	95	96	139	148	0.16	163	165	133	137	0.15
Peak HR (bpm)	108	105	158	156	0.07	182	172	144	146	0.17
V̇O_2_ (L/min)	1.30	1.17	1.13	1.00	1.08	2.13	1.98	1.17	1.14	0.14
V̇O_2_ (mL/kg/min)	15.8	14.4	16.7	14.8	3.22	29.3	27.2	14.0	13.8	0.11
V̇_E_ (L/min)	40.3	37.4	31.8	29.5	0.43	85.0	81.6	38.1	39.1	0.04
RER	0.99	0.95	0.91	0.96	0.14	1.09	1.11	1.06	1.10	1.26
RPE (6–20)	13	17	17	17	0.94	17	17	15	17	1.01
Enjoyment (1–7)	7	6	1	1	0.11	3	2	3	2	5.0

Data are presented as the mean throughout each trial, unless stated otherwise. Effect sizes are calculated using Cohen’s *d*, whereby > 0.2, >0.5, and > 0.8 represent small (black fill, white text), moderate (grey fill, black text), and large (grey fill, white text) effects, respectively. RPE was assessed using the Borg 6–20 scale (6 = *no exertion* to 20 = *maximal exertion*). Exercise enjoyment was measured using a 7-point scale (1 = *not at all* to 7 = *extremely*). DBP, diastolic blood pressure; ESCS, epidural spinal cord stimulation HR, heart rate; RER, respiratory exchange ratio; RPE, rating of perceived exertion; SBP, systolic blood pressure; SCS, spinal cord stimulation; TSCS, transcutaneous spinal cord stimulation; V̇_E_, minute ventilation; V̇CO_2_, carbon dioxide production; V̇O_2_, oxygen consumption.

## Discussion

This case series demonstrated that ESCS and TSCS improved resting cardiovascular outcomes and subsequently enhanced acute submaximal upper-body exercise performance in individuals with an upper-thoracic or cervical, chronic, motor-complete SCI. In particular, not only is this study the first to show that non-invasive TSCS can improve upper-body exercise performance but also the first to observe comparable improvements relative to ESCS.

Delivering SCS in the lower thoracic/lumbosacral region is the most commonly reported location for increasing BP at rest in individuals with SCI (Law et al., 2023). Others have identified this area as the “haemodynamic hotspot” for modulating cardiovascular control (Squair et al., 2021). Our mapping sessions with TSCS demonstrated robust haemodynamic responses upon receiving dual stimulation over the T11 and L1 spinal segments. The similar increases in predicted left ventricular contractility (with both SCS strategies) are supported by the acute improvements in contractile function with SCS reported in an animal model of ischaemic heart failure (Liu et al., 2012). However, there were seemingly greater changes in predicted TPR with ESCS in comparison to TSCS. This is perhaps due to how ESCS can target and thus activate specific sympathetic circuitry to improve vasomotor tone below the level of injury (Harkema et al., 2018; Aslan et al., 2018; Angeli et al., 2023). Such activation of sympathetic vasomotor efferents, particularly in the thoracolumbar region (Yanagiya et al., 2004), may reactivate vasoconstriction of peripheral arteries and/or splanchnic vascular beds to increase venous return and raise BP (Harkema et al., 2018). Recent reviews have discussed a mechanism known as the somato-autonomic reflex (Flett et al., 2022; Samejima et al., 2024). In short, activation of primary afferent fibres results in the excitation of spinal interneurons, which relay these input signals to sympathetic pre-, and subsequently, post-ganglionic neurons that ultimately stimulate functional changes in the target organ, which as stated is vasoconstriction. Multiple other stimulation strategies that are capable of influencing sympathetic tissues (e.g., blood vessels) may have also contributed to the subsequent improvements in exercise performance with CV-SCS and are described in a comprehensive review by Flett et al. (2022).

During exercise, the CV-SCS parameters seemingly modulated supraspinal sympathetic drive to increase resting BP and subsequently peak oxygen pulse, implying a greater peak SV (Nightingale et al., 2019) and thus cardiac output, which may be supported by no noticeable change in HR across trials. A greater peak systolic velocity and shear rate in the superficial femoral artery with CV-SCS, albeit in only two participants, complements the increases in BP and peak oxygen pulse. This potentially larger SV likely enabled a greater delivery of substrates to meet the metabolic demands of the upper-body musculature, supported the removal of waste metabolites, and ultimately prolonged the onset of fatigue (Joyner and Casey, 2015). Furthermore, the lack of change in leg muscle activity during the exercise trials suggests that the modulation of BP could be due to the reactivation of vasomotor tone alone, rather than via a skeletal muscle pump mechanism to increase venous return. Limitations in filtering TSCS artefacts from the trunk EMG signals restricted our understanding of trunk muscle activation in response to CV-SCS and whether this may have improved trunk stability and therefore exercise performance. Arm-cycling and other exercises are known to activate trunk musculature in individuals with SCI > T6 (Bjerkefors et al., 2009, 2014; Williams et al., 2020), but it is possible that SCS may further increase this activation through the recruitment of spinal pathways that innervate these muscles or through direct stimulation of muscle tissue adjacent to the stimulation site (e.g., erector spinae).

A recent finding suggests that TSCS may increase BP by simply lowering the threshold for AD (Solinsky et al., 2024). However, other TSCS studies have demonstrated its preventative and therapeutic effects on episodes of AD (Collins and DiCarlo, 2002; Sachdeva et al., 2021). The mapping used in this study to identify optimal transcutaneous electrode placement, together with cautious monitoring of cardiovascular responses, enabled a safe and controlled elevation in BP with TSCS without inducing cardiovascular-related adverse events. Importantly, this case series also included individuals with varying levels of fitness, one of which exhibited a V̇O_2peak_ on par with elite athletes with SCI (Baumgart et al., 2018). The robust improvements in exercise performance across all participants suggest that the ergogenic benefits of SCS are not limited to either sedentary or trained populations. A recent survey regarding the opinions of individuals with SCI on SCS strategies revealed that over 80% of respondents would find maintaining physical health a key benefit if they were to receive SCS, and thus, 91% would be willing to follow a specific training/rehabilitation protocol (Thorogood et al., 2023). More individuals would be interested in trying TSCS (80%) versus ESCS (61%), perhaps owing to its relatively non-invasive nature. With the eventual commercialisation of TSCS devices, there is likely to be greater community uptake by individuals with SCI, with its utilisation improving acute exercise performance and possibly eliciting greater therapeutic adaptations as part of a longitudinal intervention. The lower RPE during the exercise trials may also have an important translation to individuals using SCS to minimise fatigue during activities of daily living.

There are several considerations to this study. First, our small sample size warrants adequately powered studies to corroborate our findings that ESCS and TSCS can improve exercise performance in individuals with SCI. Second, there are calls for research to prescribe exercise intensity via traditional physiological anchors (e.g., ventilatory threshold) as the use of fixed percentages (i.e., % V̇O_2peak_, %HR_peak_) in individuals with SCI results in large inter-individual variation (Hutchinson and Goosey-Tolfrey, 2021; Hodgkiss et al., 2023). However, it is notoriously difficult to identify ventilatory thresholds in individuals with higher neurological levels of injury (Au et al., 2018; Kouwijzer et al., 2019). As such, it was difficult to identify these thresholds and subsequently prescribe a uniform exercise intensity across all four participants. Furthermore, during the familiarisation sessions we observed different responses to exercise performed at V_T1_ versus V_T2_, likely due to differences in age, aerobic capacity, and upper-extremity function. Therefore, to overcome this we prescribed a workload corresponding to *at or above* the midpoint of V_T1_ and V_T2_, allowing individualised workloads while still implementing an element of consistency across both matched pairs of participants. Future research could consider using lactate threshold (i.e., the gold standard) to prescribe individualised workloads. Third, while recent research has begun to report on novel filtering approaches to manage TSCS artefacts in EMG signals, these are only in EMG recordings from the limbs where TSCS contamination is considerably less, and often in recordings where substantial voluntary muscle activity is present (Kim et al., 2021; Andrews et al., 2023). Neither of these novel filters nor more common EMG filters were able to adequately filter TSCS artefacts from our trunk muscle recordings. As research continues to demonstrate the benefits of SCS therapies, there is a clear need for experts in the field of signal processing to develop techniques to effectively filter TSCS artefacts from nearby EMG recordings in the trunk, so we may better understand the effect of SCS on trunk muscle activity. Fourth, we acknowledge that the short period in which the TSCS intensity was increased for the SHAM-SCS trial may have resulted in some residual physiological changes at the very start of exercise, yet the large improvements in time to exhaustion with CV-SCS likely support that these effects would not have persisted throughout the SHAM-SCS trial. Finally, while our mapping data indicated a possible decrease in SV with ESCS, this is likely to be due to a limitation within the Modelflow^®^ prediction resulting from rapid alterations in haemodynamic control/stability with noisy SCS (Waldron et al., 2018).

Evidently, further work is required to tease apart the distinct mechanisms by which ESCS and TSCS alter cardiovascular output and thus contribute to improvements in upper-body exercise performance. Looking forward, researchers should consider using echocardiography to elucidate any changes in cardiac function during mapping sessions. Furthermore, given that we could only measure shear rate in two participants, adequately powered studies should explore whether increases in shear rate during exercise drive any beneficial endothelial physiological adaptations in non-active limbs in this population and whether this is influenced by SCS. The long-term effects of both ESCS and TSCS on exercise capacity and other prominent health markers are also an important avenue for future research. Finally, while there are promising data supporting the safety of both SCS strategies (Pino et al., 2022; Megía García et al., 2020), infrequent adverse events have been reported (Chalif et al., 2024) and therefore researchers should endeavour to be transparent with adverse event reporting going forward.

## Conclusion

With optimised CV-SCS parameters, both ESCS and TSCS mitigated resting hypotension and improved acute upper-body exercise performance in individuals with a cervical or upper-thoracic, motor-complete SCI. These findings have important ramifications for normalising physiological responses to exercise in a compromised clinical population. However, further study is warranted to corroborate and validate the findings reported in this case series, along with understanding the mechanisms underpinning these improvements. As TSCS is a non-invasive and relatively less expensive alternative to ESCS, it could be used as a therapeutic tool to stimulate meaningful physiological adaptions during exercise training or rehabilitation, thereby reducing the risk of chronic disease and improving health-related quality of life in individuals with SCI.

## Data Availability

The original contributions presented in the study are included in the article/Supplementary material, further inquiries can be directed to the corresponding authors.
