# Supplementary material for: Ergogenic effects of spinal cord stimulation on exercise performance following spinal cord injury
Source: Front Neurosci. 2024 Aug 29;18:1435716. doi: 10.3389/fnins.2024.1435716 (PMC11390595; doi:10.3389/fnins.2024.1435716)
Supplement: Supplementary file 1 [file Data_Sheet_1.docx]

**Supplementary material S1**

**Ergogenic effects of spinal cord stimulation on exercise performance following spinal cord injury**

**Frontiers in Neuroscience**

Daniel D. Hodgkiss, MSci ^1^, Alison M.M. Williams, MSc ^2,3^, Claire S. Shackleton, PhD ^2,4^, Soshi Samejima, DPT, PhD ^2,5^, Shane J.T. Balthazaar, PhD ^1,2,6^, Tania Lam, PhD ^2,3^, Andrei V. Krassioukov, PhD, MD ^2,4,7^*, Tom E. Nightingale, PhD ^1,2^*

**^1^** School of Sport, Exercise and Rehabilitation Sciences, University of Birmingham, UK.

**^2^** International Collaboration on Repair Discoveries (ICORD), University of British Columbia, Vancouver, British Columbia, Canada. **^3^** School of Kinesiology, University of British Columbia, Vancouver, BC, Canada. ^4^ Division of Physical Medicine and Rehabilitation, Department of Medicine, University of British Columbia, Vancouver, Canada. ^5^ Department of Rehabilitation Medicine, University of Washington, Seattle, USA. ^6^ Division of Cardiology, University of British Columbia, Vancouver General and St. Paul’s Hospital Echocardiography Department, Vancouver, BC, Canada. ^7^ GF Strong Rehabilitation Centre, Vancouver Coastal Health, Vancouver, BC, Canada

**Corresponding authors:** Andrei V. Krassioukov, PhD, MD ([andrei.krassioukov@vch.ca](mailto:andrei.krassioukov@vch.ca))

& Tom E. Nightingale PhD ([T.E.Nightingale@bham.ac.uk](mailto:T.E.Nightingale@bham.ac.uk))

| **eTable 1.** Participant demographics of the seven individuals who completed the mapping session. | | | | | | | | | |
| --- | --- | --- | --- | --- | --- | --- | --- | --- | --- |
|  | **Age (years)** | **TSI (years)** | **LOI** | **AIS** | **Height (cm)** | **Weight (kg)** | **V̇O_2peak_ (mL/kg/min)** | **Classification ^a^** | **Peak W** |
| **ESCS P1** | 24 | 3 | C6 | B | 179 | 81.7 | 16.0 | Excellent | 42 |
| **ESCS P2** | 59 | 5 | T4 | A | 177 | 68.9 | 21.3 | Good | 55 |
| **ESCS P3** | 38 | 10 | C6 | A | 172 | 83.4 | 14.2 | Good | 70 |
|  |  |  |  |  |  |  |  |  |  |
| **TSCS P1** | 40 | 19 | T4 | A | 178 | 72.6 | 31.2 | Excellent | 162 |
| **TSCS P2** | 54 | 6 | T4 | A | 173 | 80.5 | 13.9 | Fair | 66 |
| **TSCS P3** | 22 | 1 | C7 | A | 182 | 66.1 | 15.0 | Good | 59 |
| **TSCS P4** | 35 | 6 | C6 | B | 195 | 97.5 | 15.4 | Excellent | 35 |

^a^ Classifications of peak oxygen uptake (V̇O_2peak_) are based on reference fitness values for the SCI population, described by Simmons et al.^1^ AIS, American Spinal Injury Association Impairment Scale Grade; LOI, level of injury; TSI, time since injury; V̇O_2peak_, peak oxygen uptake; W, watts.

| **eTable 2.** Specific spinal cord stimulation parameters identified as optimizing cardiovascular control (CV-SCS) for all seven participants who completed the mapping session. | | | | | |
| --- | --- | --- | --- | --- | --- |
|  | **Electrode locations** | **Time since implant** | **Frequency** | **Pulse width** | **Current** |
| **ESCS P1** | T10 – T12 | 7 months | 30 Hz | 500 μs | 6.1 mA |
| **ESCS P2** | T11 – L1 | 3 years | 300 Hz | 380 μs | 3.6 mA |
| **ESCS P3** | T11 – T12 | 5 years | 35 Hz | 300 μs | 6 V |
|  |  |  |  |  |  |
| **TSCS P1** | T11 – L1 | - | 30 Hz burst + 10 kHz carrier | 1.0 ms | 39 mA |
| **TSCS P2** | T11 – L1 | - | 30 Hz burst + 10 kHz carrier | 1.0 ms | 78 mA |
| **TSCS P3** | T11 – L1 | - | 30 Hz burst + 10 kHz carrier | 1.0 ms | 39 mA |
| **TSCS P4** | T11 – L1 | - | 30 Hz burst + 10 kHz carrier | 1.0 ms | 65 mA |

| **eTable 3.** Cardiovascular outcomes at baseline (without SCS) and with cardiovascular-optimised spinal cord stimulation (CV-SCS) during the mapping session. | | | | | | | | | |  |
| --- | --- | --- | --- | --- | --- | --- | --- | --- | --- | --- |
|  | **Mean SBP (mmHg)** | | **Mean HR (bpm)** | | **Peak SV (mL)** | | **Peak dP/dt_max_ (mmHg/s)** | | **Mean TPR (mmHg/s/mL)** | |
|  | **Baseline** | **CV-SCS** | **Baseline** | **CV-SCS** | **Baseline** | **CV-SCS** | **Baseline** | **CV-SCS** | **Baseline** | **CV-SCS** |
| **ESCS P1** | 123 | 134 | 59 | 65 | 106 | 99 | 942 | 1104 | 0.95 | 1.19 |
| **ESCS P2** | 118 | 143 | 62 | 71 | 74 | 73 | 652 | 746 | 1.23 | 1.67 |
| **ESCS P3** | 70 | 113 | 87 | 65 | 79 | 137 | 423 | 720 | 0.52 | 0.61 |
|  |  |  |  |  |  |  |  |  |  |  |
| **TSCS P1** | 108 | 129 | 73 | 64 | 98 | 116 | 955 | 1208 | 0.73 | 0.93 |
| **TSCS P2** | 117 | 146 | 98 | 83 | 68 | 97 | 961 | 1228 | 0.82 | 0.85 |
| **TSCS P3** | 124 | 146 | 65 | 57 | 149 | 138 | 1120 | 1825 | 0.58 | 0.84 |
| **TSCS P4** | 77 | 112 | 71 | 64 | 118 | 145 | 827 | 1233 | 0.58 | 0.51 |

dP/dt_max_, left ventricular cardiac contractility; HR, heart rate; SBP, systolic blood pressure; SV, stroke volume; TPR, total peripheral resistance.

**References**

1. Simmons OL, Kressler J, Nash MS. Reference fitness values in the untrained spinal cord injury population. *Arch Phys Med Rehabil*. 2014;95(12):2272-2278.
