# Supplementary material for: Ergogenic effects of spinal cord stimulation on exercise performance following spinal cord injury
Source: Front Neurosci. 2024 Aug 29;18:1435716. doi: 10.3389/fnins.2024.1435716 (PMC11390595; doi:10.3389/fnins.2024.1435716)
Supplement: Supplementary file 2 [file Data_Sheet_2.docx]

**Supplementary material S2**

**Ergogenic effects of spinal cord stimulation on exercise performance following spinal cord injury**

**Frontiers in Neuroscience**

Daniel D. Hodgkiss, MSci ^1^, Alison M.M. Williams, MSc ^2,3^, Claire S. Shackleton, PhD ^2,4^, Soshi Samejima, DPT, PhD ^2,5^, Shane J.T. Balthazaar, PhD ^1,2,6^, Tania Lam, PhD ^2,3^, Andrei V. Krassioukov, PhD, MD ^2,4,7^*, Tom E. Nightingale, PhD ^1,2^*

**^1^** School of Sport, Exercise and Rehabilitation Sciences, University of Birmingham, UK.

**^2^** International Collaboration on Repair Discoveries (ICORD), University of British Columbia, Vancouver, British Columbia, Canada. **^3^** School of Kinesiology, University of British Columbia, Vancouver, BC, Canada. ^4^ Division of Physical Medicine and Rehabilitation, Department of Medicine, University of British Columbia, Vancouver, Canada. ^5^ Department of Rehabilitation Medicine, University of Washington, Seattle, USA. ^6^ Division of Cardiology, University of British Columbia, Vancouver General and St. Paul’s Hospital Echocardiography Department, Vancouver, BC, Canada. ^7^ GF Strong Rehabilitation Centre, Vancouver Coastal Health, Vancouver, BC, Canada

**Corresponding authors:** Andrei V. Krassioukov, PhD, MD ([andrei.krassioukov@vch.ca](mailto:andrei.krassioukov@vch.ca))

& Tom E. Nightingale PhD ([T.E.Nightingale@bham.ac.uk](mailto:T.E.Nightingale@bham.ac.uk))

**
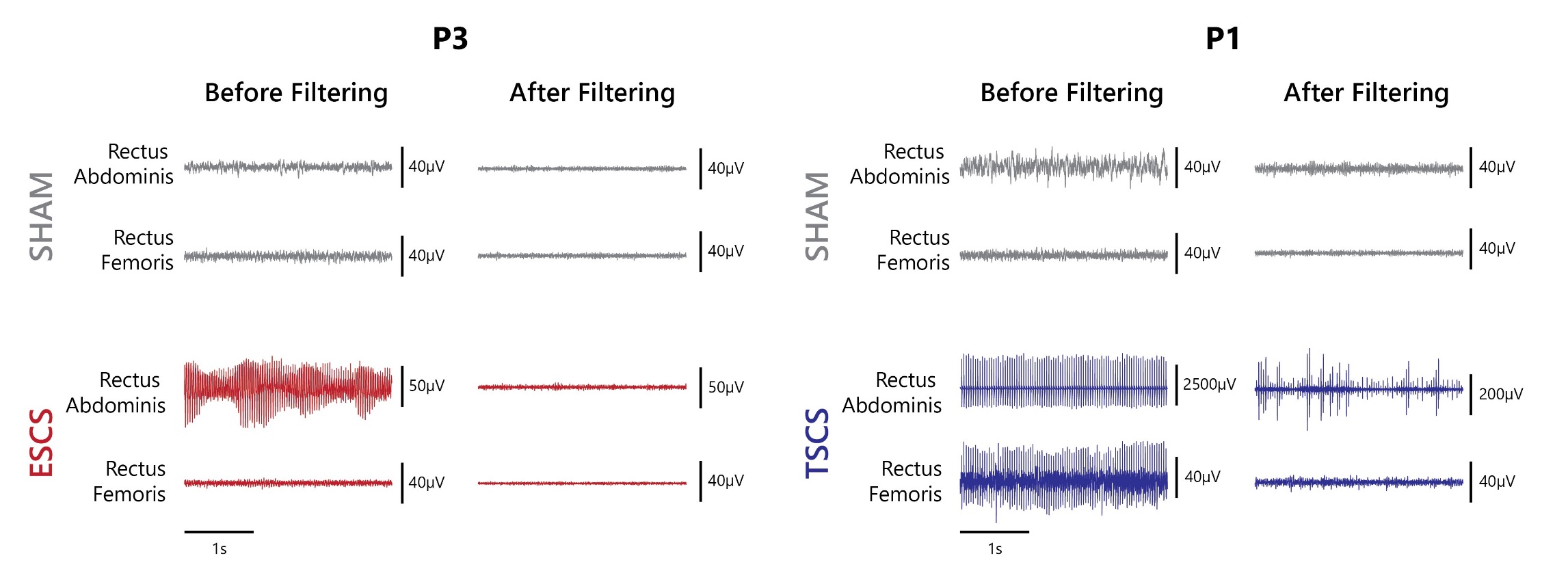
Figure 1.** Surface electromyography (EMG) sensors (Trigno, Delsys Inc, Natick, USA) were affixed bilaterally to the rectus abdominis (RA), external obliques (EO), erector spinae at the L4 level (ES), rectus femoris (RF), biceps femoris (BF), tibialis anterior (TA), and the medial head of gastrocnemius (MG). All EMG data were collected at 2000 Hz and stored for offline analysis. During the mapping session for selection of CV-SCS and SHAM-SCS, 10s recordings were taken after 1 minute of exposure to a given stimulation intensity/configuration. During the exercise trials, a 60-s recording was taken with participants seated at rest prior to the application of ESCS or TSCS, and then 60-s recordings were taken every 5-min during exercise. EMG data were analysed using custom MATLAB routines (Mathworks Inc., Natick, USA). All EMG data were high pass filtered at 10Hz with an eight-order Butterworth filter and then low-pass filtered at 300 Hz via the same filter. Notch filters were applied at harmonics of 30Hz to reduce contamination of the 30Hz CV-SCS. We then took the average of the root mean square (RMS) of each signal. For each participant, we then calculated the mean RMS value for each muscle while the participant was at rest, and during each of the recordings during the exercise. After which, an average profile of the trunk (RA, EO, ES) and leg (RF, BF, TA, MG) was calculated for at rest and across the exercise recordings for each visit, for each participant. The standard deviation of the averaged trunk and leg EMG was also taken across all time points in order to calculate effect sizes. We explored many approaches for filtering the TSCS artifacts in the trunk EMG signals recorded from P1 and P2 in their CV-SCS sessions. Previously published filters for removing TSCS artifacts in EMG signals were not effective^1,2^, and in viewing the results of different filters in both the time and frequency domains, we found the best approach was to use a low and high-pass Butterworth filter with additional notch filters at harmonics of 30Hz (described above). While this approach was successful in removing all ESCS artifacts and TSCS artifacts in the legs, residual TSCS artifacts remained in the trunk muscle recordings. This figure presents the results of this approach in one participant who received ESCS (P3) and one who received TSCS (P1) in recordings from an abdominal muscle (rectus abdominis) and leg muscle (rectus femoris). These data were collected during the SHAM-SCS (grey) and CV-SCS visits (ESCS in red; TSCS in blue). Our filter was successful in filtering the ESCS artifacts from participants who received ESCS. However, the amplitudes of these artifacts were relatively small in the trunk, and there were limited or no artifacts in the leg recordings. In comparison, the filter was successful in removing the artifacts in the leg recordings from participants who received TSCS, but some artifacts remained in the trunk. While the filter did improve the signal quality of the trunk recordings, the remaining contamination would result in inaccurate quantification of muscle activity, and as such we decided not to report the trunk results for this study.

**References**

[1] Kim M, Moon Y, Hunt J, et al. A Novel Technique to Reject Artifact Components for Surface EMG Signals Recorded During Walking With Transcutaneous Spinal Cord Stimulation: A Pilot Study. Front Hum Neurosci. 2021;15:660583.

[2] Andrews B, Karem A, Harkema SJ, Rouffet DM. Artifact Adaptive Ideal Filtering of EMG Signals Contaminated by Spinal Cord Transcutaneous Stimulation. IEEE Trans Neural Syst Rehabil Eng. 2023;31:3047-3054.
