# Supplementary material for: Ergogenic effects of spinal cord stimulation on exercise performance following spinal cord injury
Source: Front Neurosci. 2024 Aug 29;18:1435716. doi: 10.3389/fnins.2024.1435716 (PMC11390595; doi:10.3389/fnins.2024.1435716)
Supplement: Supplementary file 3 [file Data_Sheet_3.docx]

**Supplementary Figure S1**

**Ergogenic effects of spinal cord stimulation on exercise performance following spinal cord injury**

**Frontiers in Neuroscience**

Daniel D. Hodgkiss, MSci ^1^, Alison M.M. Williams, MSc ^2,3^, Claire S. Shackleton, PhD ^2,4^, Soshi Samejima, DPT, PhD ^2,5^, Shane J.T. Balthazaar, PhD ^1,2,6^, Tania Lam, PhD ^2,3^, Andrei V. Krassioukov, PhD, MD ^2,4,7^*, Tom E. Nightingale, PhD ^1,2^*

**^1^** School of Sport, Exercise and Rehabilitation Sciences, University of Birmingham, UK.

**^2^** International Collaboration on Repair Discoveries (ICORD), University of British Columbia, Vancouver, British Columbia, Canada. **^3^** School of Kinesiology, University of British Columbia, Vancouver, BC, Canada. ^4^ Division of Physical Medicine and Rehabilitation, Department of Medicine, University of British Columbia, Vancouver, Canada. ^5^ Department of Rehabilitation Medicine, University of Washington, Seattle, USA. ^6^ Division of Cardiology, University of British Columbia, Vancouver General and St. Paul’s Hospital Echocardiography Department, Vancouver, BC, Canada. ^7^ GF Strong Rehabilitation Centre, Vancouver Coastal Health, Vancouver, BC, Canada

**Corresponding authors:** Andrei V. Krassioukov, PhD, MD ([andrei.krassioukov@vch.ca](mailto:andrei.krassioukov@vch.ca))

& Tom E. Nightingale PhD ([T.E.Nightingale@bham.ac.uk](mailto:T.E.Nightingale@bham.ac.uk))


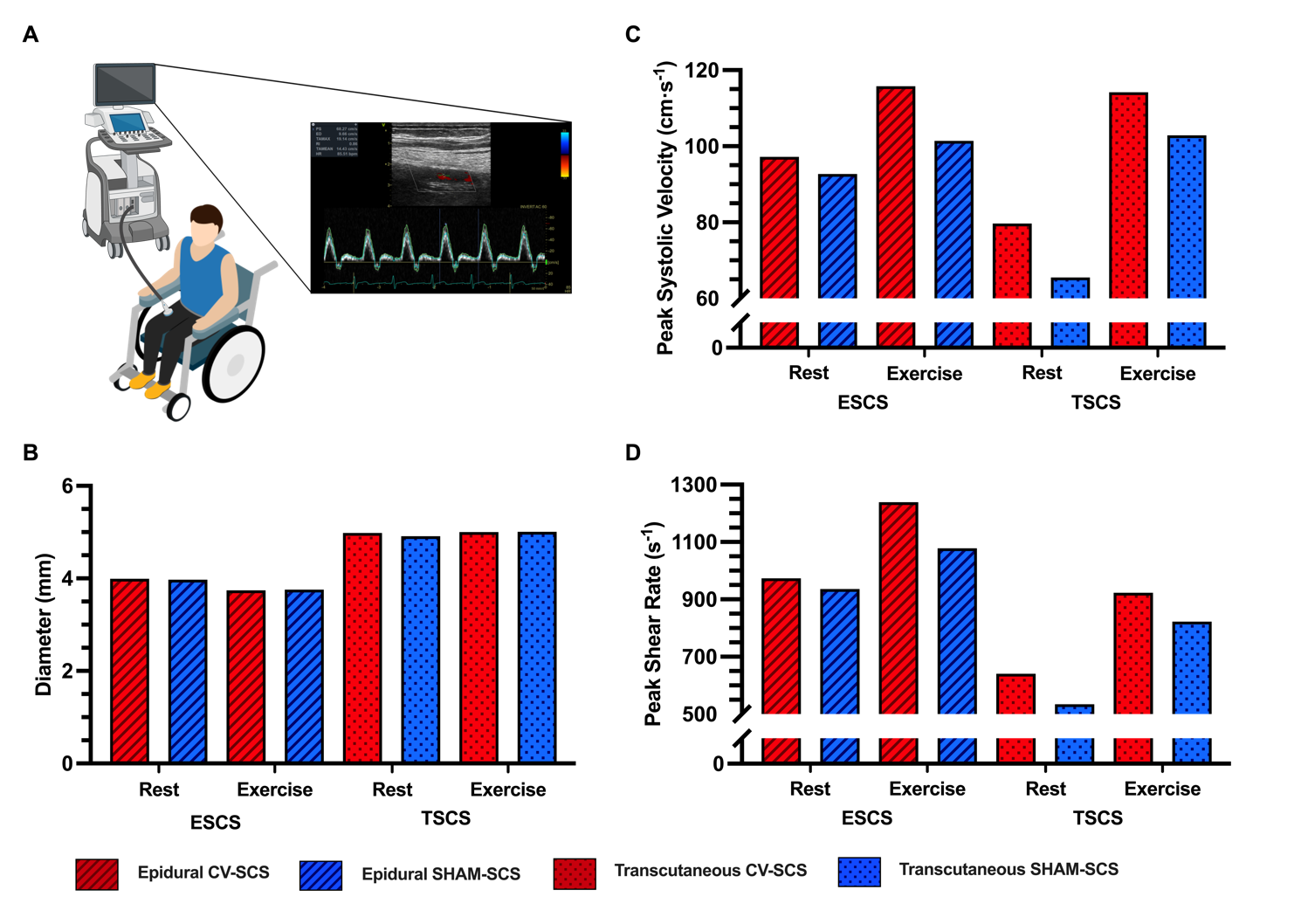


**Suppl Figure S1. (A)** Superficial femoral artery blood flow and vessel diameter was assessed using a 12L-RS Doppler linear vascular probe (5.2-13MHz, Vivid 7, GE Healthcare, Mississauga, ON) without stimulation at rest and every 5-min with CV-SCS or SHAM-SCS during the trial, with the participants seated upright. Data was collected in one matched SCS pair (ESCS P2 and TSCS P2) only, due to limited availability of the sonographer. The transducer had a fixed insonation angle of 60° on the participants’ right leg. A final measurement was taken upon the end of the trial. Following acquisition, images were stored offline in Digital Imaging and Communications in Medicine format for analysis. Artery images were measured from five consecutive cycles (excluding artifacts) in a video loop and then averaged. Vessel diameter was assessed using B-mode ultrasound imaging. Mean blood velocity, defined as the average velocity of the enveloping Doppler spectrum during the entire cardiac cycle, was calculated and averaged from five consecutive cycles. Mean blood flow was calculated from the product of the arterial cross-sectional area (πr2) and the average velocity. **(B-D)** Resting and exercise (mean) parameters with CV-SCS and SHAM-SCS. During exercise, peak systolic velocity was greater with CV-SCS relative to SHAM-SCS in both participants. Peak shear rate during exercise was greater with CV-SCS, relative to SHAM-SCS, in both participants but larger with ESCS in comparison to TSCS. Vessel diameters were comparable, thus demonstrating reproducibility. CV-SCS, cardiovascular optimized spinal cord stimulation; SHAM-SCS, sham spinal cord stimulation.
